# Evaluation of Sleep Disorders and Quality of Life in Patients With Mycosis Fungoides

**DOI:** 10.1002/cnr2.70421

**Published:** 2025-12-29

**Authors:** Seyed AmirReza Mohammadi, Mozhdeh Sepaskhah, Arvin Hedayati, Ladan Dastgheib, Soheila Khodakarim, Mohammad Amin Gholami

**Affiliations:** ^1^ School of Medicine Shiraz University of Medical Sciences Shiraz Iran; ^2^ Department of Dermatology, School of Medicine, Molecular Dermatology Research Center Shiraz University of Medical Sciences Shiraz Iran; ^3^ Department of Psychiatry Shiraz University of Medical Sciences Shiraz Iran; ^4^ Molecular Dermatology Research Center & Department of Dermatology, Shiraz University of Medical Sciences Shiraz Iran; ^5^ Department of Biostatistics Shiraz University of Medical Sciences Shiraz Iran; ^6^ Student Research Committee Shiraz University of Medical Sciences Shiraz Iran

**Keywords:** cutaneous T‐cell lymphoma, mycosis Fungoides, PSQI, quality of life, sleep disorders

## Abstract

**Background:**

Mycosis fungoides (MF) is the most common cutaneous T‐cell lymphoma, presenting with variable clinical features that can be pruritic. Pruritus and the overall disease burden may disrupt sleep and diminish quality of life; yet, sleep disturbances in MF remain under‐investigated.

**Aims:**

To evaluate sleep quality and quality of life in MF patients compared with healthy controls.

**Methods and Results:**

In a cross‐sectional, case–control study at a referral clinic in Shiraz, Iran, 78 MF patients (TNMB stages IA–IIIA) and 76 age‐ and sex‐matched controls completed the Pittsburgh Sleep Quality Index (PSQI), the 12‐Item Short Form Survey (SF‐12), and the Hospital Anxiety and Depression Scale (HADS). MF patients had significantly poorer sleep quality, with 62.8% reporting poor sleep quality compared to 27.6% of controls (*p* < 0.001). Among MF patients, poorer sleep quality was significantly associated with greater pruritus intensity, more advanced disease stage, and a higher modified severity‐weighted assessment tool score (mSWAT) (all *p* < 0.05). In contrast, histopathological subtype and treatment modality showed no significant association. MF patients exhibited higher rates of moderate‐to‐severe anxiety (34.6% vs. 15.8%) and depression (30.7% vs. 14.6%) (*p* < 0.05). Physical health scores on the SF‐12 were significantly lower in the MF group (*p* < 0.01), whereas the decrease in mental health scores did not reach significance.

**Conclusion:**

MF is associated with significant sleep dysfunction, psychological distress, and impaired physical quality of life. Pruritus intensity, disease stage, and cutaneous disease burden are key contributors to poor sleep quality. Larger studies are warranted to further elucidate the extent and mechanisms of sleep disturbance in this population.

## 1 Introduction

Mycosis fungoides (MF) is the most prevalent form of cutaneous T‐cell lymphoma [1]. This disease presents as various skin lesions, including patches, plaques, tumors, or erythroderma, often accompanied by itching, which can significantly impact patients' quality of life [2]. The clinical heterogeneity of MF, reflected in its diverse manifestations, poses challenges in both diagnosis and management. The exact cause of MF is unknown; however, genetic and epigenetic alterations may play a role in the disease [3, 4].

The incidence of MF in the United States and Europe is approximately 6 cases per million people per year, accounting for about 4% of non‐Hodgkin lymphomas. Recent meta‐analytic data, including more than 16 000 CTCL cases, confirm that MF incidence has remained low yet is gradually increasing worldwide, with estimates of 6–7 cases per million annually [5, 6, 7]. Studies of mycosis fungoides (MF) in Iran are limited in both numbers and cases. A study conducted in Isfahan revealed that the average age of Iranian MF patients was unexpectedly lower at 41 years old, and the male‐to‐female ratio was 1:1.2—a notable contrast to the US ratio of 1.3:1 [6, 8].

Health‐related quality of life (HRQoL) is a major concern in mycosis fungoides and cutaneous T‐cell lymphoma. Multiple cross‐sectional and survey‐based studies, including cohorts of several hundred patients, have demonstrated substantial impairment in physical, emotional, and social functioning in this population. In a large CTCL cohort with predominantly MF, Demierre et al. reported marked limitations in daily activities, emotional well‐being, and sleep, highlighting the broad psychosocial burden of disease [9]. More recently, Shinohara et al. and other groups have confirmed that HRQoL is significantly reduced in MF/Sezary syndrome, particularly in patients with more advanced stages, greater symptom burden, or severe pruritus [10]. Another study focusing on the early and mild stages of MF, involving 30 patients, suggested limited effects on the quality of life, anxiety, and depression during these initial stages [11]. A systematic review evaluating the quality of life in MF patients suggests that MF exhibits a broad spectrum of symptoms that, depending on disease severity, significantly reduce the quality of life, ranging from moderate to severe [12].

Sleep disorders adversely affect diverse physiological processes, bodily functions, and quality of life, correlating with conditions such as obesity, diabetes, hypertension, and depression [13, 14, 15, 16, 17]. Research on psoriasis, another skin condition, indicates a significant association with sleep disorders. For example, a meta‐analysis of 15 studies involving 1274 patients with psoriasis confirmed that psoriasis significantly impairs sleep, with patients exhibiting markedly poorer sleep quality and a higher risk of sleep disorders compared to controls [18]. Risk factors for sleep disturbances in psoriasis include itching, pain, depression, anxiety, and disease severity [19, 20]. Given that both MF and psoriasis share similarities in inflammatory processes and itching, investigating sleep disorders in MF patients seems reasonable [21].

Prior work on pruritic dermatoses has demonstrated that nocturnal itch and related factors disrupt sleep and are associated with fatigue, emotional distress, and poorer quality of life (QoL) [18, 22, 23]. Despite these observations, at the time of devising the manuscript, to our knowledge, no MF‐specific studies had systematically evaluated sleep using validated instruments—particularly in comparison with matched controls—highlighting the need for the present study.

However, despite the growing literature on HRQoL in MF and CTCL, the specific interplay among MF, sleep disorders, and quality of life has not been systematically examined using validated sleep measures, compared with matched controls. Itching, a common symptom in MF patients, can disrupt sleep and impair daytime functioning and overall quality of life [21]. As previously noted, MF presents a relatively low prevalence both globally and in Iran. This study addresses key gaps in the existing literature on MF by offering novel insights into its understudied aspects. Accordingly, it aims to evaluate sleep disorders and quality of life in patients with MF.

## 2 Methods

### 2.1 Study Design

This cross‐sectional, case–control study was conducted at the Cutaneous T‐cell lymphoma Clinic within a referral dermatology clinic in Shiraz, Iran, from October 2022 to September 2023. The study aimed to evaluate two primary components: sleep disorders and quality of life in patients diagnosed with MF. Additionally, depression and anxiety disorders were assessed to provide a more comprehensive understanding of the psychological effects of the disease.

There were 78 patients with a confirmed diagnosis of MF attending the clinic during the study period. Controls were 76 age‐ and sex‐matched individuals without MF, selected from the general population attending other clinics within the Imam Reza Clinical Complex as friends or family members of other patients. Participants were enrolled using consecutive sampling to ensure representativeness.

### 2.2 Data Gathering

Clinical data for the patients were collected from complete histories, physical examinations, and three specific questionnaires. For diagnosing mycosis fungoides (MF), we adopted the diagnostic algorithm recommended by the International Society for Cutaneous Lymphoma (ISCL), which integrates clinical, histopathological, immunopathological, and molecular criteria. A diagnosis of MF is confirmed if a patient amasses at least 4 points according to this algorithm [24, 25]. Pruritus severity was assessed with the verbal rating scale (VRS) [26]. Data Collection involved patients completing validated questionnaires under supervision to ensure the completeness and accuracy of their responses. In this study, three questionnaires were utilized to collect the desired information:

1. Pittsburgh Sleep Quality Index (PSQI): This questionnaire assesses sleep disturbances by measuring individuals' perceptions of sleep quality over the past 4 weeks. It comprises seven components designed to cover a broad spectrum of sleep quality aspects. Each component is scored from 0 (no problem) to 3 (severe problem), and the scores are summed to derive an overall sleep quality score. A total score exceeding 5 indicates poor sleep quality [27]. The PSQI's validity and reliability are well documented across international studies, and its Persian adaptation has been specifically validated for use in the Iranian population [28].
2. 12‐Item Short Form Survey (SF‐12): This questionnaire is an efficient, shortened version of the SF‐36, designed to evaluate health‐related quality of life across various populations. The outcomes are compiled into Physical and Mental Component Scores (PCS and MCS, respectively), scaled from 0 to 100, with higher scores reflecting better health status. The validity and reliability of the SF‐12 have been confirmed in various international studies, and its Persian version has been validated for use in the Iranian population [29, 30].
3. Hospital Anxiety and Depression Scale (HADS): This questionnaire is a brief, self‐administered tool designed for identifying depression and anxiety. The maximum score is 21, with score interpretations as follows: 0–7 (none), 8–10 (mild), 11–14 (moderate), and 15–21 (severe) for anxiety or depression. It is widely recognized and validated, including its Persian version, which has been assessed for validity and reliability in the Iranian population [31, 32].

Treatment modalities were recorded as binary variables for each type (e.g., topical therapy: yes/no), allowing for multiple concurrent treatments per patient to reflect real‐world clinical practice. However, this approach did not include a formal analysis of specific combinations due to sample size constraints.

### 2.3 Inclusion and Exclusion Criteria

The inclusion criteria for the MF group were as follows: age between 18 and 80 years, an ISCL diagnostic algorithm score of at least 4, and provision of written informed consent. Conversely, patients were excluded if they did not provide informed consent.

The control group was selected from the general population and matched to the MF group by age and gender. Individuals in the control group had to be between 18 and 80 years old and provide written informed consent. Those who did not provide consent or had a diagnosis of mycosis fungoides were excluded.

### 2.4 Endpoints and Assessments

1. Primary endpoint: Sleep quality, assessed by the PSQI (prevalence of poor sleep defined as PSQI > 5), comparing MF versus control.

2. Secondary endpoints: Health‐related quality of life (SF‐12 PCS/MCS) and psychological symptoms (HADS‐Anxiety, HADS‐Depression), comparing MF versus control.

3. Exploratory endpoints (within MF group): Associations of pruritus intensity (assessed by Verbal Rating Scale [VRS]), cutaneous disease burden (measured by modified Severity Weighted Assessment Tool [mSWAT]), clinical stage/subtype, current treatment categories, and disease duration with poor sleep (PSQI > 5).

### 2.5 Statistical Analysis

Quantitative data were summarized using means and standard deviations, while qualitative data were presented as frequencies and percentages. Data analysis was performed using SPSS software, version 18 (SPSS Inc., Chicago, USA).

The normality of continuous variables was assessed using the Shapiro–Wilk test. For variables that followed a normal distribution, descriptive statistics were presented as means and standard deviations, and parametric tests were employed for inferential analyses. For non‐normally distributed variables, medians and interquartile ranges were reported, and non‐parametric tests were utilized accordingly.

To compare continuous variables between two independent groups, the independent *t*‐test was used for normally distributed data, while the Mann–Whitney U test was used for data that did not meet the normality assumptions. Categorical variables were analyzed using the Chi‐square test to evaluate associations between groups.

When comparing continuous variables across three or more independent groups, such as the severity of pruritus and burning sensation, MF stages, and MF subtypes, the Kruskal‐Wallis H test was used. This nonparametric test was selected because the data were nonnormal.

To examine the relationships between continuous variables—namely, sleep quality scores, physical component scores, and mental component scores—the Spearman rank correlation coefficient was calculated. A *p*‐value of less than 0.05 was considered statistically significant for all analyses.

In designing the study, the novel objective of investigating sleep disorders in patients with mycosis fungoides prompted us to base our sample size calculation on data from sleep disorders in patients with psoriasis. This decision was made based on dermatological similarities and the shared symptom of pruritus between the two conditions. We input the data into the NCSS PASS application, setting the Type I error rate (α) at 0.05 and the statistical power (1‐β) at 0.8 [33]. Consequently, a sample size of 71 patients was determined for the case group.

## 3 Results

A total of 78 patients were enrolled in the MF group, and 76 in the control group. As shown in Table 1, there were no statistically significant differences in age or gender distribution between the two groups (*p* > 0.05). Table A1 summarizes the clinical characteristics of patients with MF.

**TABLE 1:** Comprehensive comparison of demographic characteristics, sleep quality, anxiety, depression, and quality of life between mycosis fungoides patients and healthy controls.

Variables		Groups		*p*
		Case (*n* = 78)	Control (*n* = 76)	
Age (years), Mean ± SD		42.5 ± 13.6	41.9 ± 13.5	0.489
Sex, *n* (%)	Male	30 (38.5%)	30 (39.5%)	0.898
	Female	48 (61.5%)	46 (60.5%)	
PSQI result, *n* (%)	Good sleep†	29 (37.2%)	55 (72.4%)	< 0.001^a^
	Poor sleep ‡	49 (62.8%)	21 (27.6%)	
HADS anxiety, *n* (%)	No anxiety§	36 (46.2%)	48 (63.2%)	0.012^a^
	Mild anxiety	15 (19.2%)	16 (21.1%)	
	Moderate anxiety	18 (23.1%)	8 (10.5%)	
	Severe anxiety	9 (11.5%)	4 (5.3%)	
HADS depression, *n* (%)	No depression	35 (44.9%)	49 (64.5%)	0.008^a^
	Mild depression	19 (24.4%)	16 (21.1%)	
	Moderate depression	21 (26.9%)	9 (11.8%)	
	Severe depression	3 (3.8%)	2 (2.6%)	
Physical component score, Mean (SD)		44.7 (9.8)¶	49.4 (8.6)	0.003^a^
Mental component score, Mean (SD)		42 (10.5)	45.3 (8.9)	0.057

Abbreviations: HADS = Hospital Anxiety and Depression Scale; PSQI = Pittsburgh Sleep Quality Index; SD = Standard Deviation.

^a^ *p* < 0.05 indicates statistical significance; †Good Sleep Quality = PSQI score ≤ 5; ‡Poor Sleep Quality = PSQI score > 5; §Categories are based on standard HADS cutoffs: 0–7 (No), 8–10 (Mild), 11–14 (Moderate), 15–21 (Severe); ¶ 12‐Item Short Form Health Survey component scores range from 0 to 100, with higher scores indicating better physical or mental health.

MF patients exhibited significantly poorer sleep quality than healthy controls, as measured by the Pittsburgh Sleep Quality Index (PSQI) (*p* < 0.001). Specifically, 62.8% of MF patients reported poor sleep quality, compared to only 27.6% of healthy controls (Table 1).

**FIGURE 1:**
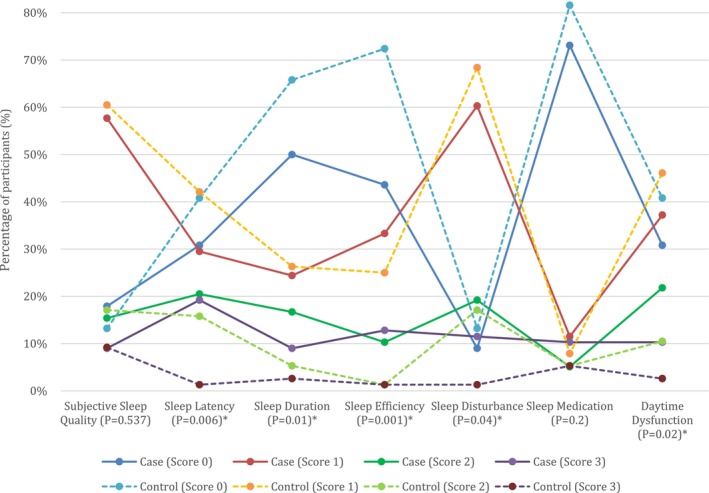
Distribution of Pittsburgh Sleep Quality Index (PSQI) component scores in mycosis fungoides patients and healthy controls. Lines represent the percentage of participants in the respective group (Case or Control) who scored 0, 1, 2, or 3 for each of the seven PSQI components. A score of 0 indicates no sleep quality issues in that aspect, while higher scores indicate poorer sleep quality. *p*‐values are provided for each component, with significant differences (*p* < 0.05) marked by an asterisk (*).

In our analysis of PSQI components (Figure 1), MF patients exhibited significantly poorer scores in five out of seven components compared to healthy controls: Sleep Latency (*p* = 0.006), Sleep Duration (*p* = 0.013), Sleep Efficiency (*p* = 0.001), Sleep Disturbance (*p* = 0.046), and Daytime Dysfunction (*p* = 0.02). The remaining two components—Subjective Sleep Quality and Use of Sleep Medication—did not differ significantly (*p* = 0.53 and 0.2, respectively). Each component is presented in detail in Table A2.

In Table 1, we also present the Hospital Anxiety and Depression Scale (HADS) results comparing MF patients and healthy controls. Among MF patients, 46.2% were classified as having no anxiety compared to 63.2% of controls. In contrast, moderate and severe anxiety were observed in 23.1% and 11.5% of MF patients, respectively, versus 10.5% and 5.3% among controls, with the overall anxiety distribution differing significantly between groups (*p* = 0.012). Regarding depression, only 44.9% of MF patients were classified as having no depression compared to 64.5% of healthy controls. Additionally, mild and moderate depression were more prevalent in MF patients (24.4% and 26.9%, respectively) than in controls (21.1% and 11.8%, respectively), yielding a statistically significant difference in depression levels (*p* = 0.008). These findings indicate that MF patients are more likely to experience clinically significant anxiety and depression than their healthy counterparts.

Quality of life was assessed using the 12‐Item Short Form Survey (SF‐12). The physical component score was significantly lower in MF patients (44.7 ± 9.8) than in healthy controls (49.4 ± 8.6; *p* = 0.003), indicating greater physical impairment among patients. Although the mental component score was also lower in MF patients (42.0 ± 10.5) relative to controls (45.3 ± 8.9), this difference did not reach statistical significance (*p* = 0.057). These results suggest that while MF patients experience significant physical limitations, the impact on mental health may be less pronounced, warranting further investigation.

As shown in Table 2, sleep disturbance among patients with MF was significantly associated with pruritus severity (*p* = 0.047), advanced disease stage (*p* = 0.040), and higher mSWAT scores (*p* = 0.039). In contrast, neither the histopathological subtype of MF nor the treatment modality had a significant impact on sleep quality.

**TABLE 2:** Association of pruritus, clinical characteristics, and treatment modalities with sleep quality in patients with mycosis fungoides.

Category	Subcategory	Good sleep†	Poor sleep	Total‡	*p*
VRS, *n* (%)	No itch	15 (48.4%)	16 (51.6%)	31	0.047^a^
	Mild itch	10 (35.7%)	18 (64.3%)	28	
	Moderate itch	4 (25.0%)	12 (75.0%)	16	
	Severe itch	0 (0.0%)	3 (100.0%)	3	
MF stage, *n* (%)§	I A	19 (46.3%)	22 (53.7%)	41	0.040^a^
	I B	6 (25.0%)	18 (75.0%)	24	
	II A	1 (20.0%)	4 (80.0%)	5	
	II B	1 (100.0%)	0 (0.0%)	1	
	III A	0 (0.0%)	4 (100.0%)	4	
MF subtype, *n* (%)	Classic	21 (35.6%)	38 (64.4%)	59	0.769
	Erythrodermic	0 (0.0%)	3 (100.0%)	3	
	Poikilodermatous	1 (33.3%)	2 (66.7%)	3	
	Hypopigmented	3 (50.0%)	3 (50.0%)	6	
	Papular	1 (50.0%)	1 (50.0%)	2	
	Folliculotropic	1 (50.0%)	1 (50.0%)	2	
Treatment, *n* (%)¶	No TOPICAL	7 (33.3%)	14 (66.7%)	21	0.764
	Topical	20 (37.0%)	34 (63.0%)	54	
	No phototherapy	7 (26.9%)	19 (73.1%)	26	0.233
	Phototherapy	20 (40.8%)	29 (59.2%)	49	
	No methotrexate	25 (37.3%)	42 (62.7%)	67	0.493
	Methotrexate	2 (25.0%)	6 (75.0%)	8	
	No interferon	23 (39.7%)	35 (60.3%)	58	0.233
	Interferon	4 (23.5%)	13 (76.5%)	17	
	No retinoid	19 (35.2%)	35 (64.8%)	54	0.814
	Retinoid	8 (38.1%)	13 (61.9%)	21	
mSWAT score	Mean (SD)	13.5 (22.3)	23.0 (28.3)	—	0.039^a^
	25th percentile	0.2	2.0	—	
	50th percentile	3.0	7.5	—	
	75th percentile	15.0	35.7	—	

*Note:* Missing patient data were three cases each for Stage, Subtype, and mSWAT. Percentages are based on available cases.

Abbreviations: MF = mycosis fungoides; mSWAT = modified severity weighted assessment tool; SD = standard deviation; VRS = Verbal Rating Scale for pruritus.

^a^ *p* < 0.05 indicates statistical significance; †Good Sleep Quality = PSQI score ≤ 5 and Poor Sleep Quality = PSQI score > 5; ‡Total patients in each subcategory; §TNMB Staging; ¶ Treatment modalities were analyzed individually as binary variables, with patients potentially counted in multiple categories if receiving combined regimens.

**FIGURE A1:**
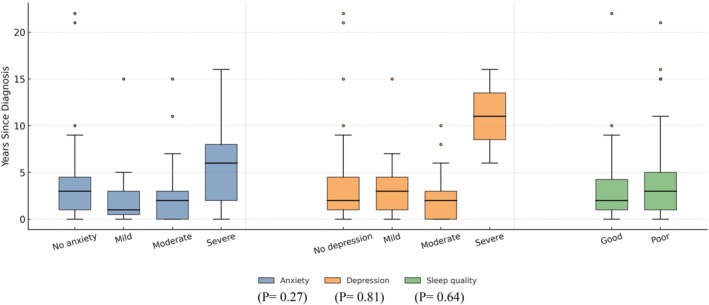
Time Since Diagnosis of MF Stratified by Anxiety, Depression, and Sleep Quality. MF = Mycosis fungoides; *p* = *p*‐value. The number of valid participants was 77 (after exclusion of missing data). Boxes represent the interquartile range (IQR) with the median indicated by the line within the box; whiskers extend to 1.5 × IQR beyond the box edges; individual points denote outliers. Anxiety and depression were categorized using the Hospital Anxiety and Depression Scale (HADS) subscales, with cutoffs as follows: 0–7 (none), 8–10 (mild), 11–14 (moderate), 15–21 (severe) for both HADS‐A (anxiety) and HADS‐D (depression). Poor sleep quality was defined as a global Pittsburgh Sleep Quality Index (PSQI) score of greater than 5. The three plots are displayed side by side with a common *y*‐axis for comparison.

In an exploratory analysis (Figure A1), we examined whether time since MF diagnosis might influence patients' anxiety, depression, or sleep quality status. The median time since diagnosis did not appear to differ meaningfully among patients when stratified by anxiety (*p* = 0.276), depression (*p* = 0.810), or sleep quality (*p* = 0.642). These exploratory findings suggest that longer disease duration may not be a significant determinant of psychological distress or sleep disturbances in MF.

## 4 Discussion

The results of our study reveal distinct differences between patients with mycosis fungoides and the normal population across multiple domains, providing a foundation for understanding the broader implications of this condition. MF patients demonstrated markedly poorer sleep quality. Notably, five of seven PSQI components—sleep latency, duration, efficiency, disturbances, and daytime dysfunction—were significantly impaired in MF patients, whereas subjective sleep quality and medication use did not differ, suggesting objective metrics may better capture sleep deficits in this population. Further, MF patients exhibited heightened psychological burden, with higher rates of moderate‐to‐severe anxiety and depression compared to controls. Quality‐of‐life assessments using the SF‐12 further underscored these findings, revealing significantly lower physical health scores in MF patients, while the difference in mental health scores approached but did not reach statistical significance. These findings collectively underscore the multifactorial impact of MF, extending beyond cutaneous manifestations to disrupt sleep, mental health, and physical well‐being.

Previous studies have evaluated quality of life in patients with MF, and a systematic review found that even early‐stage MF is associated with mild‐to‐moderate reductions in quality of life [12, 34]. Our study, conducted in an outpatient setting with predominantly early‐stage MF patients, supports these findings, revealing a significantly lower SF‐12 Physical Component Score in patients with MF compared to controls, indicating impaired physical health. Additionally, our results demonstrated a higher prevalence of anxiety and depression among MF patients compared to controls. These observations align with prior research, which has highlighted the critical role of anxiety and depression in reducing quality of life in this population. Furthermore, a cohort study has suggested a correlation between MF and these psychological conditions, reinforcing the interplay between mental health and disease burden in MF [35]. Recent cross‐sectional data from Asian MF/SS cohorts similarly demonstrate substantial psychological, social, and economic burden across disease stages, further supporting our findings of heightened anxiety and depression in MF patients [36].

At the start of our study, sleep disturbances in MF patients had not been systematically evaluated or reported in the literature. However, pruritus—previously identified as the most frequent and bothersome symptom in MF [12, 34]—may directly contribute to sleep disturbances by disrupting rest. Given the coexistence of pruritus with elevated levels of anxiety, depression, and reduced quality of life in MF patients, we hypothesized that sleep disturbances might also be more prevalent in this disease. Our results confirmed this hypothesis, showing significantly poorer sleep quality in patients with MF.

Within the MF cohort, the drivers of sleep impairment appeared to be disease‐ and symptom‐related rather than treatment‐related. Poorer PSQI scores were significantly associated with higher pruritus severity, more advanced clinical stages, and a greater cutaneous tumor burden, as measured by mSWAT. In contrast, neither the histopathological subtype of MF nor the treatment modality (analyzed as individual binary variables) showed a significant association with sleep quality in this exploratory analysis. However, the lack of detailed assessment of therapeutic combinations may limit this finding. These observations reinforce the central role of itch and overall disease activity in disrupting sleep, suggesting that controlling pruritus and limiting cutaneous involvement may offer the greatest leverage for improving sleep quality, regardless of the MF subtype or the specific therapy being used.

Previous literature shows that pruritic skin diseases such as psoriasis, atopic dermatitis, and lichen planus often disrupt sleep due to nocturnal itching, leading to daytime fatigue, emotional distress, and reduced quality of life [21]. In psoriasis, for example, most patients experience sleep disturbances at least 1 day per month, with itching, pain, depression, anxiety, and disease severity identified as key risk factors [19, 37, 38]. Consistent with findings in other pruritic dermatoses, sleep quality in our MF cohort was significantly compromised, with a significant correlation to pruritus intensity and overall cutaneous disease burden. Notably, contemporary HRQoL frameworks in MF have emphasized the need to assess sleep disturbance as a distinct contributor to overall disease burden, a gap our study directly addresses [39].

This study highlights previously unexplored aspects of MF while confirming its known adverse effects on psychological well‐being and quality of life. Notably, our findings highlight the high prevalence of sleep disturbances in MF patients, underscoring the importance of comprehensive screening beyond reliance on subjective assessments of sleep quality. We encourage clinicians to proactively identify and address these sleep disturbances as part of an integrated management approach to reduce the overall disease burden.

Further research is needed to fully elucidate the relationship between sleep disturbances and MF. Future studies would benefit from larger sample sizes that include inpatient populations and a greater representation of advanced disease stages—an area in which this study was limited—to provide a more comprehensive understanding of the impact of MF on sleep quality.

### 4.1 Strengths and Limitations

A key strength of this study is its comprehensive evaluation of multiple aspects of mycosis fungoides, a condition with a complex and often under‐researched clinical profile. While previous studies have assessed the quality of life in MF patients, none have simultaneously examined the interplay between depression, anxiety, and sleep quality at the time of the study. This unique approach provides valuable insights into the combined impact of these factors on patients' overall well‐being.

Additionally, this study is the first to evaluate sleep quality in patients with MF, an aspect that has previously been overlooked and considered of less importance. By addressing this gap, the study underscores the importance of screening for and managing sleep disturbances in this patient population.

The study was conducted at a Specialized Referral Center for mycosis fungoides, where patient clinical data were carefully collected and largely complete. An extensive face‐to‐face collection of questionnaire data ensured minimal missing data.

Since this study was conducted in an outpatient setting, most participants had milder stages of mycosis fungoides that did not require hospitalization. Consequently, data on patients with advanced stages of the disease is limited, and the study may not fully represent the experience of those with more severe forms of MF. Additionally, the majority of patients had already been diagnosed and were undergoing treatment, leading to a scarcity of data on newly diagnosed cases or those not receiving treatment. These factors may have influenced the study's ability to detect certain significant correlations or patterns.

Recruiting controls as companions of outpatient clinic patients constitutes a clinic‐based convenience sample, which may introduce selection bias (e.g., caregiver burden or unmeasured comorbidities) and potentially inflate reported sleep disturbances or psychological symptoms in the control group [40, 41]. Consequently, generalizability to the broader population may be limited; future studies should consider community‐based sampling to mitigate this bias.

Additionally, concomitant non‐dermatologic medications were not reliably ascertained (owing to participant non‐disclosure/recall limitations), and detailed comorbidity data were not systematically collected; residual confounding by these factors remains possible and may influence between‐group differences in sleep and psychological outcomes. Because we relied on univariate analyses and our sample size was limited due to correlated predictors, we did not fit multivariable models, so the independence of effects cannot be established. Future studies should prospectively capture medication/comorbidity data to enable adjustment and, with adequate power, test whether pruritus remains associated with poor sleep after adjustment for mSWAT, HADS anxiety/depression, age, sex, and treatment pattern.

## 5 Conclusion

In conclusion, patients with mycosis fungoides had markedly poorer sleep quality, more frequent anxiety and depression, and lower physical health‐related quality of life compared with age‐ and sex‐matched controls. More than half of the MF patients were classified as poor sleepers, and sleep disruption affected multiple dimensions, including sleep latency, duration, efficiency, disturbances, and daytime functioning. Clinically, our results suggest that routine care for patients with MF should extend beyond skin examination to include structured assessment of sleep, pruritus, and psychological symptoms using validated instruments. Targeted management of itch and anxiety/depression, together with optimized control of cutaneous disease, may help to improve both sleep quality and quality of life. Future studies with larger, more diverse MF populations, including patients at more advanced stages and across different care settings, are needed to confirm these findings and evaluate interventions specifically aimed at improving sleep and overall well‐being in this patient group.

### 5.1 Ethical Considerations

All data were treated as confidential. Informed consent was obtained in written form from all participants, who were informed of their right to withdraw from the study at any time without any consequences. The study received approval from the Ethics Committee of Shiraz University of Medical Sciences (IR.SUMS.MED.REC.1401.371), which oversees research at affiliated clinical sites, including the study location. It was conducted in accordance with the Declaration of Helsinki and relevant national guidelines and regulations. Additionally, all collected questionnaires were coded with unique identifiers instead of patient names to ensure privacy and were securely stored, accessible only to authorized research personnel.

## Author Contributions

**Mozhdeh Sepaskhah:** conceptualization, methodology, investigation, supervision, resources, writing – original draft, writing – review and editing. **Seyed AmirReza Mohammadi:** writing – original draft, investigation.

## Funding

This work was supported by the Vice‐Chancellor for Research, Shiraz University of Medical Sciences, 26594.

## Conflicts of Interest

The authors declare no conflicts of interest.

## Data Availability

The data that support the findings of this study are available from the corresponding author upon reasonable request.

## Appendix A

**TABLE A1:** Clinical characteristics of patients with mycosis fungoides.

Category	Subcategory	Case (*n* = 78)/value
MF stage, *n* (%)†	IA	41 (54.7%)
	IB	24 (32.0%)
	IIA	5 (6.7%)
	IIB	1 (1.3%)
	IIIA	4 (5.3%)
MF Subtype, *n* (%)	Classic	59 (78.6%)
	Erythrodermic	3 (4.0%)
	Poikilodermatous	3 (4.0%)
	Hypopigmented	6 (8.0%)
	Papular	2 (2.7%)
	Folliculotropic	2 (2.7%)
mSWAT score	Mean (SD)	19.6 (26.6)
	25th Percentile	1
	50th Percentile	4.5
	75th Percentile	33
Treatment, *n* (%)‡	Topical	54 (72.0%)
	Phototherapy	49 (65.3%)
	Methotrexate	8 (10.7%)
	Interferon	17 (22.7%)
	Retinoid	21 (28.0%)
	No Treatment	4 (5.1%)
VRS, *n* (%)	No itch	31 (39.7%)
	Mild itch	28 (35.9%)
	Moderate itch	16 (20.5%)
	Severe itch	3 (3.8%)

*Note:* Missing patient data were three cases each for Stage, Subtype, and mSWAT. Percentages are based on available cases. †TNMB Staging; ‡ Treatment type the patient is currently receiving.

Abbreviations: MF = mycosis fungoides; mSWAT = modified severity weighted assessment tool; SD = standard deviation; VRS = Verbal Rating Scale for pruritus.

**TABLE A2:** Pittsburgh Sleep Quality Index component score in MF patients and healthy controls.

Sleep quality score		Groups		*p*‐value
		Case (*n* = 78)	Control (*n* = 76)	
Subjective sleep quality, *n* (%)	0	14 (17.9%)	10 (13.2%)	0.537
	1	45 (57.7%)	46 (60.5%)	
	2	12 (15.4%)	13 (17.1%)	
	3	7 (9%)	7 (9.2%)	
Sleep latency, *n* (%)	0	24 (30.8%)	31 (40.8%)	0.006^a^
	1	23 (29.5%)	32 (42.1%)	
	2	16 (20.5%)	12 (15.8%)	
	3	15 (19.2%)	1 (1.3%)	
Sleep duration, *n* (%)	0	39 (50%)	50 (65.8%)	0.013^a^
	1	19 (24.4%)	20 (26.3%)	
	2	13 (16.7%)	4 (5.3%)	
	3	7 (9%)	2 (2.6%)	
Sleep efficiency, *n* (%)	0	34 (43.6%)	55 (72.4%)	< 0.001^a^
	1	26 (33.3%)	19 (25%)	
	2	8 (10.3%)	1 (1.3%)	
	3	10 (12.8%)	1 (1.3%)	
Sleep disturbance, *n* (%)	0	7 (9%)	10 (13.2%)	0.046^a^
	1	47 (60.3%)	52 (68.4%)	
	2	15 (19.2%)	13 (17.1%)	
	3	9 (11.5%)	1 (1.3%)	
Use of sleep medication, *n* (%)	0	57 (73.1%)	62 (81.6%)	0.200
	1	9 (11.5%)	6 (7.9%)	
	2	4 (5.1%)	4 (5.3%)	
	3	8 (10.3%)	4 (5.3%)	
Daytime dysfunction, *n* (%)	0	24 (30.8%)	31 (40.8%)	0.02^a^
	1	29 (37.2%)	35 (46.1%)	
	2	17 (21.8%)	8 (10.5%)	
	3	8 (10.3%)	2 (2.6%)	

Abbreviation: MF = mycosis fungoides.

^a^ Indicates *p* < 0.05.

## References

[1] A. C. Hristov , T. Tejasvi , and R. A. Wilcox , “Mycosis Fungoides and Sezary Syndrome: 2019 Update on Diagnosis, Risk‐Stratification, and Management,” American Journal of Hematology 94, no. 9 (2019): 1027–1041. 31313347 10.1002/ajh.25577

[2] B. Engin , A. S. Keçici , A. Uzun , and M. Yalçın , “Psychiatric Comorbidity, Depression, and Anxiety Levels and Quality of Life of the Patients With Mycosis Fungoides,” Dermatologic Therapy 33, no. 6 (2020): e13922. 32594601 10.1111/dth.13922

[3] C. P. Tensen , K. D. Quint , and M. H. Vermeer , “Genetic and Epigenetic Insights Into Cutaneous T‐Cell Lymphoma,” Blood 139, no. 1 (2022): 15–33. 34570882 10.1182/blood.2019004256

[4] A. Erdemir and O. Erdem , “Etiopathogenesis of Mycosis Fungoides,” Turkish Journal of Dermatology 19 (2025): 1–6.

[5] M. Sant , C. Allemani , C. Tereanu , et al., “Incidence of Hematologic Malignancies in Europe by Morphologic Subtype: Results of the HAEMACARE Project,” Blood 116, no. 19 (2010): 3724–3734. 20664057 10.1182/blood-2010-05-282632

[6] Z. R. Cai , M. L. Chen , M. A. Weinstock , Y. H. Kim , R. A. Novoa , and E. Linos , “Incidence Trends of Primary Cutaneous T‐Cell Lymphoma in the US From 2000 to 2018: A SEER Population Data Analysis,” JAMA Oncology 8, no. 11 (2022): 1690–1692. 36048455 10.1001/jamaoncol.2022.3236 PMC9437821

[7] G. Dobos , A. Pohrt , C. Ram‐Wolff , et al., “Epidemiology of Cutaneous T‐Cell Lymphomas: A Systematic Review and Meta‐Analysis of 16,953 Patients,” Cancers (Basel) 12, no. 10 (2020): 2921. 33050643 10.3390/cancers12102921 PMC7600606

[8] F. Fatemi Naeini , B. Abtahi‐Naeini , H. Sadeghiyan , M. A. Nilforoushzadeh , J. Najafian , and M. Pourazizi , “Mycosis Fungoides in Iranian Population: An Epidemiological and Clinicopathological Study,” Journal of Skin Cancer 2015 (2015): 306543. 25694829 10.1155/2015/306543 PMC4324921

[9] M. F. Demierre , S. Gan , J. Jones , and D. R. Miller , “Significant Impact of Cutaneous T‐Cell Lymphoma on Patients' Quality of Life: Results of a 2005 National Cutaneous Lymphoma Foundation Survey,” Cancer 107, no. 10 (2006): 2504–2511. 17048251 10.1002/cncr.22252

[10] M. M. Shinohara , H. M. Mahurin , E. Tarabadkar , et al., “Health‐Related Quality of Life in Cutaneous T‐Cell Lymphoma: A Cross‐Sectional Survey Study,” Skin Health and Disease 1, no. 3 (2021): e45. 35663135 10.1002/ski2.45 PMC9060149

[11] O. Segal , N. Trumper , F. Pavlotsky , G. Goldzweig , and A. Barzilai , “Illness Perception, Coping, and Quality of Life in Early‐Stage Mycosis Fungoides,” Anais Brasileiros de Dermatologia 96, no. 1 (2021): 27–33. 33279315 10.1016/j.abd.2020.05.008 PMC7838113

[12] R. Ottevanger , S. van Beugen , A. W. M. Evers , R. Willemze , M. H. Vermeer , and K. D. Quint , “Quality of Life in Patients With Mycosis Fungoides and Sezary Syndrome: A Systematic Review of the Literature,” Journal of the European Academy of Dermatology and Venereology 35, no. 12 (2021): 2377–2387. 34331819 10.1111/jdv.17570 PMC9291074

[13] J. E. Gangwisch , “A Review of Evidence for the Link Between Sleep Duration and Hypertension,” American Journal of Hypertension 27, no. 10 (2014): 1235–1242. 24778107 10.1093/ajh/hpu071 PMC4229731

[14] Y. Zeng , J. Wu , J. Yin , J. Chen , S. Yang , and Y. Fang , “Association of the Combination of Sleep Duration and Sleep Quality With Quality of Life in Type 2 Diabetes Patients,” Quality of Life Research 27, no. 12 (2018): 3123–3130. 30030675 10.1007/s11136-018-1942-0

[15] J.‐P. Chaput , A. W. McHill , R. C. Cox , et al., “The Role of Insufficient Sleep and Circadian Misalignment in Obesity,” Nature Reviews Endocrinology 19, no. 2 (2023): 82–97. 10.1038/s41574-022-00747-7 PMC9590398 36280789

[16] D. A. Nôga , M. EdMeS , A. P. Pacheco , et al., “Habitual Short Sleep Duration, Diet, and Development of Type 2 Diabetes in Adults,” JAMA Network Open 7, no. 3 (2024): e241147. 38441893 10.1001/jamanetworkopen.2024.1147 PMC10915681

[17] K. Hosseini , H. Soleimani , K. Tavakoli , et al., “Association Between Sleep Duration and Hypertension Incidence: Systematic Review and Meta‐Analysis of Cohort Studies,” PLoS One 19, no. 7 (2024): e0307120. 39008468 10.1371/journal.pone.0307120 PMC11249221

[18] M. Guo , J. Su , S. Zheng , and B. Chen , “Sleep in Psoriasis: A Meta‐Analysis,” Journal of Psychosomatic Research 176 (2024): 111543. 37956475 10.1016/j.jpsychores.2023.111543

[19] B. Halioua , C. Chelli , L. Misery , J. Taieb , and C. Taieb , “Sleep Disorders and Psoriasis: An Update,” Acta Dermato‐Venereologica 102 (2022): adv00699. 35191513 10.2340/actadv.v102.1991 PMC9574693

[20] L. Misery , “Sleep Disturbance and Psoriasis,” Journal of the European Academy of Dermatology and Venereology 36, no. 5 (2022): 633. 35416364 10.1111/jdv.18081

[21] I. Podder , H. Mondal , and G. Kroumpouzos , “Nocturnal Pruritus and Sleep Disturbance Associated With Dermatologic Disorders in Adult Patients,” International Journal of Women's Dermatology 7, no. 4 (2021): 403–410. 10.1016/j.ijwd.2021.02.010 PMC8484989 34632036

[22] K. Kaaz , J. C. Szepietowski , and Ł. Matusiak , “Influence of Itch and Pain on Sleep Quality in Atopic Dermatitis and Psoriasis,” Acta Dermato‐Venereologica 99, no. 2 (2019): 175–180. 30307027 10.2340/00015555-3065

[23] Y. S. Chang and B. L. Chiang , “Sleep Disorders and Atopic Dermatitis: A 2‐Way Street?,” Journal of Allergy and Clinical Immunology 142, no. 4 (2018): 1033–1040. 30144472 10.1016/j.jaci.2018.08.005

[24] N. Pimpinelli , E. A. Olsen , M. Santucci , et al., “Defining Early Mycosis Fungoides,” Journal of the American Academy of Dermatology 53, no. 6 (2005): 1053–1063. 16310068 10.1016/j.jaad.2005.08.057

[25] E. Olsen , E. Vonderheid , N. Pimpinelli , et al., “Revisions to the Staging and Classification of Mycosis Fungoides and Sezary Syndrome: A Proposal of the International Society for Cutaneous Lymphomas (ISCL) and the Cutaneous Lymphoma Task Force of the European Organization of Research and Treatment of Cancer (EORTC),” Blood 110, no. 6 (2007): 1713–1722. 17540844 10.1182/blood-2007-03-055749

[26] A. Reich , M. Heisig , N. Q. Phan , et al., “Visual Analogue Scale: Evaluation of the Instrument for the Assessment of Pruritus,” Acta Dermato‐Venereologica 92, no. 5 (2012): 497–501. 22102095 10.2340/00015555-1265

[27] D. J. Buysse , C. F. Reynolds, 3rd , T. H. Monk , S. R. Berman , and D. J. Kupfer , “The Pittsburgh Sleep Quality Index: A New Instrument for Psychiatric Practice and Research,” Psychiatry Research 28, no. 2 (1989): 193–213. 2748771 10.1016/0165-1781(89)90047-4

[28] J. Farrahi Moghaddam , N. Nakhaee , V. Sheibani , B. Garrusi , and A. Amirkafi , “Reliability and Validity of the Persian Version of the Pittsburgh Sleep Quality Index (PSQI‐P),” Sleep & Breathing 16, no. 1 (2012): 79–82. 21614577 10.1007/s11325-010-0478-5

[29] A. Montazeri , M. Vahdaninia , S. J. Mousavi , and S. Omidvari , “The Iranian Version of 12‐Item Short Form Health Survey (SF‐12): Factor Structure, Internal Consistency and Construct Validity,” BMC Public Health 9 (2009): 341. 19758427 10.1186/1471-2458-9-341 PMC2749829

[30] J. E. Ware , M. Kosinski , and S. D. Keller , “A 12‐Item Short‐Form Health Survey: Construction of Scales and Preliminary Tests of Reliability and Validity,” Medical Care 34, no. 3 (1996): 220–233. 8628042 10.1097/00005650-199603000-00003

[31] A. Montazeri , M. Vahdaninia , M. Ebrahimi , and S. Jarvandi , “The Hospital Anxiety and Depression Scale (HADS): Translation and Validation Study of the Iranian Version,” Health and Quality of Life Outcomes 1 (2003): 14. 12816545 10.1186/1477-7525-1-14 PMC161819

[32] A. S. Zigmond and R. P. Snaith , “The Hospital Anxiety and Depression Scale,” Acta Psychiatrica Scandinavica 67, no. 6 (1983): 361–370. 6880820 10.1111/j.1600-0447.1983.tb09716.x

[33] P. Jensen , C. Zachariae , L. Skov , and R. Zachariae , “Sleep Disturbance in Psoriasis: A Case‐Controlled Study,” British Journal of Dermatology 179, no. 6 (2018): 1376–1384. 29704428 10.1111/bjd.16702

[34] H. M. Holahan , R. S. Farah , S. Fitz , et al., “Health‐Related Quality of Life in Patients With Cutaneous T‐Cell Lymphoma?,” International Journal of Dermatology 57, no. 11 (2018): 1314–1319. 30074622 10.1111/ijd.14132

[35] E. Hodak , S. Lessin , R. Friedland , et al., “New Insights Into Associated Comorbidities in Patients With Cutaneous T‐Cell Lymphoma (Mycosis Fungoides),” Acta Dermato‐Venereologica 93, no. 4 (2013): 451–455. 23303582 10.2340/00015555-1496

[36] Y. Shi , J. Sun , H. Sun , et al., “Economic Burden and Health‐Related Quality of life in Chinese Patients With Mycosis Fungoides and Sézary Syndrome,” Cancer Pathogenesis and Therapy 3, no. 5 (2025): 434–440. 40923030 10.1016/j.cpt.2025.01.005 PMC12414272

[37] K. Callis Duffin , B. Wong , E. J. Horn , and G. G. Krueger , “Psoriatic Arthritis Is a Strong Predictor of Sleep Interference in Patients With Psoriasis,” Journal of the American Academy of Dermatology 60, no. 4 (2009): 604–608. 19167780 10.1016/j.jaad.2008.10.059

[38] J. Nowowiejska , A. Baran , M. Lewoc , P. Grabowska , T. W. Kaminski , and I. Flisiak , “The Assessment of Risk and Predictors of Sleep Disorders in Patients With Psoriasis‐A Questionnaire‐Based Cross‐Sectional Analysis,” Journal of Clinical Medicine 10, no. 4 (2021): 664. 33572270 10.3390/jcm10040664 PMC7916004

[39] C. Asare , J. Chen , P. Naidoo , et al., “Defining Core Concepts for Health‐Related Quality of Life for Patients With Mycosis Fungoides and Sezary Syndrome: A Systematic Literature Review,” JEADV Clinical Practice 4, no. 4 (2025): 907–919.

[40] K. C. Lee , J. J. Yiin , S. H. Lu , and Y. F. Chao , “The Burden of Caregiving and Sleep Disturbance Among Family Caregivers of Advanced Cancer Patients,” Cancer Nursing 38, no. 4 (2015): E10–E18. 10.1097/NCC.0000000000000166 24978619

[41] Q. Chen , L. Terhorst , A. Lowery‐Allison , et al., “Sleep Problems in Advanced Cancer Patients and Their Caregivers: Who Is Disturbing Whom?,” Journal of Behavioral Medicine 43, no. 4 (2020): 614–622. 31435891 10.1007/s10865-019-00088-3 PMC7035154

